# Prevalence and sociodemographic risk factors of rhinitis in pregnancy: A cross-sectional study from Saudi Arabia

**DOI:** 10.1097/MD.0000000000046609

**Published:** 2025-12-19

**Authors:** Hanan I. Almuzaini, Rasil H. Almughamisi, Shahad A. Aljohani, Danah A. Alnajjar, Aseel A. Alamri, Thikra A. Alsenani, Maram A. Alhazmi, Samah O. Alfahl

**Affiliations:** a Department of Otorhinolaryngology, Head and Neck Surgery; General and Specialized Surgery Department, College of Medicine, Taibah University, Al-Madinah, Saudi Arabia; b College of Medicine, Taibah University, Al-Madinah, Saudi Arabia; c Department of Family and Community Medicine and Medical Education, College of Medicine, Taibah University, Al-Madinah, Saudi Arabia.

**Keywords:** pregnancy rhinitis, prevalence, Saudi Arabia, severity, sociodemographic factors

## Abstract

This is the second study from Saudi Arabia, after a decade-long gap, that focuses on pregnancy rhinitis (PR). PR is a common, yet under-reported and under-diagnosed condition among pregnant Saudi women. This study aimed to assess the prevalence of PR and its association with sociodemographic factors in a cohort of Saudi women. We conducted a cross-sectional questionnaire-based study from August 2023 to February 2025 of pregnant women attending Ohud and Maternity and Children’s Hospitals in Medina, Saudi Arabia. The questionnaire collected sociodemographic data and information on nasal symptoms and their severity. The prevalence of PR was calculated as the percentage of women affected. We analyzed the associations between PR and sociodemographic factors using Chi-square and Fisher exact tests, with *P* ≤ .05, as statistically significant. We included pregnant women aged ≥ 18 years and excluded those with a prepregnancy history of allergic rhinitis or sinusitis. Among 386 pregnant women, 115 (29.8%) had PR. Of these, 73% reported moderate-to-severe symptoms. PR prevalence significantly declined with increasing education levels (*P* < .001) but was higher among employed women (*P* = .050) and those with chronic diseases (*P* = .028). PR is highly prevalent among pregnant Saudi women, with most cases classified as moderate to severe. Obstetricians and gynecologists, in collaboration with ENT specialists, should assess nasal symptoms early in pregnancy and educate patients about PR and its management.

## 1. Introduction

Rhinitis is an inflammation and irritation of the nasal mucosa, characterized by nasal symptoms such as stuffiness or obstruction of the nasal passage, rhinorrhea, sneezing, itching, and post-nasal discharge.^[1]^ Pregnancy rhinitis (PR) can be defined as “nasal congestion for 6 weeks or more during pregnancy, in the absence of upper respiratory tract infection, and recovery within 2 weeks post-delivery.^[2,3]^

Although PR is quite common, affecting nearly one-third of all pregnancies, it remains underreported and underdiagnosed.^[4]^ However, PR has recently gained attention due to its frequent association with snoring and obstructive sleep apnea during pregnancy.^[1,5]^ Past evidence has also shown a link between PR and gestational hypertension, intrauterine growth retardation, and lower Apgar scores in neonates.^[1]^ Bothersome symptoms accompany PR and adversely affect the quality of life of pregnant women.^[6,7]^ Allergic rhinitis is a common condition that has a major impact on quality of life and other health problems.^[8]^

The association between pregnancy rhinitis (PR) and variables, such as parity, maternal age, and preconception atopy, was investigated in a recent cross-sectional study by Alyahya et al in Al-Ahsa, Saudi Arabia. Sneezing was the most common symptom of PR and was shown to be prevalent in 39% of the 316 women surveyed. While parity had no discernible impact, this study found significant correlations between PR and maternal age at first pregnancy, family history of allergic rhinitis, and preexisting nasal symptoms. These results confirm the clinical significance of PR in the Saudi population and highlight the need for more comprehensive regional data.^[9]^

During our literature search, we identified a notable gap in regional data from Saudi Arabia. Only one study by Albahkaly et al, published almost a decade ago, examined the prevalence of PR in a cohort of 260 pregnant women from Riyadh, Saudi Arabia. In this study, the prevalence was 31.2%.^[10]^ Despite this relatively high prevalence, no subsequent studies have explored PR in other regions of the Kingdom, leaving a significant gap in the nationally representative data. Given the potential maternal and neonatal complications associated with PR, updated evidence is urgently required to inform clinical practice and public health planning. Hence, it is necessary and relevant to gather fresh evidence on the prevalence of PR in a cohort of pregnant women in Saudi Arabia.

## 2. Methodology

### 2.1. Study design and objectives

This cross-sectional survey-based study was conducted among pregnant women in Medina, Saudi Arabia. The primary objective was to assess the prevalence and severity of PR in this cohort and identify the demographic risk factors associated with the occurrence of rhinitis in pregnancy.

### 2.2. Study duration

The study was conducted from August 2023 to February 2025, involving pregnant women who attended gynecological and obstetric consultations at Ohud Hospital and Maternity and Children’s Hospital in Medina.

### 2.3. Study questionnaire

A structured and validated questionnaire was developed to evaluate PR prevalence and severity.^[11]^ This questionnaire included relevant demographic parameters, such as age, marital status (whether separated or married), education level, career status, monthly income, smoking status, body mass index, preexisting chronic diseases, number of children, type of pregnancy, and history of miscarriages. In addition, we assessed the severity of rhinitis using the Nasal Obstruction Symptom Evaluation (NOSE) score, which was incorporated into a structured questionnaire. We further evaluated the severity of individual rhinitis symptoms using a Likert scale, as detailed in the Statistical Analysis section.

### 2.4. Ethical approvals

Written informed consent was obtained from all pregnant participants before administering the questionnaire. Ethical approval for this study was obtained from the Scientific Research Ethics Committee of the College of Medicine Research Centre, Taibah University, on February 7, 2024. All procedures were conducted in accordance with the institutional ethical standards. All data were kept confidential and stored in an electronic repository accessible only to authorized study personnel.

### 2.5. Eligibility criteria

Pregnant women above the age of 18 years who willingly consented to participate and completed the questionnaire were included. We excluded women with a history of allergic rhinitis or sinusitis and those who did not complete the study questionnaire.

### 2.6. Sample size calculation

A total of 46,125 pregnant women visited these clinics for consultation based on recent data obtained from various antenatal clinics in Medina. We considered this the total available population of interest and used the Epi-Info software to calculate the required sample size as 385, with a 95% confidence interval (CI) and a 5% margin of error.

### 2.7. Statistical analysis

Descriptive statistics were used to summarize participants’ sociodemographic characteristics and pregnancy data. We expressed quantitative variables, such as prevalence, along with other categorical demographic variables, such as frequency (*N*) and percentages (%).

Chi-square and Fisher exact tests were used to examine associations between PR and the various demographic factors included in the study, considering a *P*-value ≤ .05 as statistically significant. We derived severity scores for nasal and breathing-related symptoms from the participants’ responses to the questionnaire.

Participants rated each symptom on a Likert scale (0 = no symptoms, 1 = mild, 2 = moderate, and 3 = severe), with higher values indicating greater symptom severity. The Mean Severity Score for each symptom was calculated using the following formula: mean severity score = (sum of individual symptom scores)/ (total number of participants). all statistical analyses were conducted using IBM SPSS Statistics, version 29.0.0. The relationship between clinical and demographic factors and the occurrence of PR was evaluated using a logistic regression analysis. Odds ratios (ORs) with 95% CIs were calculated for every factor.

## 3. Results

The study sample comprised 386 pregnant women who fulfilled the eligibility criteria. The majority of the participants were 26 to 35 years of age and married. Although most had either completed high school or graduated, most were unemployed. A significant portion of the women had a monthly income of <5000 Saudi Riyals; however, more than 50% of the study participants fell into the moderate-to-high income bracket of 5000–10,000 Saudi Riyals (SAR). Most of the study cohort were nonsmokers, and nearly 75% were either overweight or obese. Almost one-fourth of all study participants reported suffering from chronic disorders, the most common being diabetes, asthma, and anemia. Most of the participants were mothers of children aged > 2 years. The majority of the participants had no previous history of miscarriage. Table 1 summarizes the sociodemographic characteristics of the study cohort.

**Table 1:** Sociodemographic parameters of study participants (n = 386).

Demographic factors	Categories	Frequency N (%)
Age	18–25 yr	47 (12.2%)
	26–30 yr	123 (31.9%)
	31–35 yr	112 (29.0%)
	>35 yr	104 (26.9%)
Education level	Uneducated to middle	40 (10.4%)
	High school	120 (31.1%)
	Diploma	24 (6.2%)
	Bachelor’s and postgraduate	202 (52.3%)
Career status	Unemployed	313 (81%)
	Employee	73 (18.9%)
	Student	16 (4.1%)
Monthly income	<5000 SAR	179 (46.4%)
	5000–10,000 SAR	123 (31.9%)
	>10,000 SAR	84 (21.8%)
Smoker	No	378 (97.9%)
	Yes	8 (2.1%)
BMI	Underweight	4 (1.0%)
	Normal	94 (24.4%)
	Overweight	112 (29.0%)
	Obese Class 1 and Class 2	176 (45.6%)
Chronic diseases	No	300 (77.7%)
	Yes	86 (22.3%)
No. of children	0	77 (19.9%)
	1–3	222 (57.5%)
	4 and more	87 (22.6%)
Age of your last child	<1 yr	7 (1.8%)
	1 yr	19 (4.9%)
	2 yr	63 (16.3%)
	>2 yr	223 (57.8%)
Pregnancy type	Single	376 (97.4%)
	Twin	10 (2.6%)
Pregnancy trimester	First	89 (23%)
	Second	197 (51%)
	Third	100 (26%)
No. of miscarriage	0	258 (66.8%)
	1–2	92 (23.8%)
	≥3	36 (9.3%)

BMI = Body mass index, SAR = Saudi Riyals.

**Figure 1.**
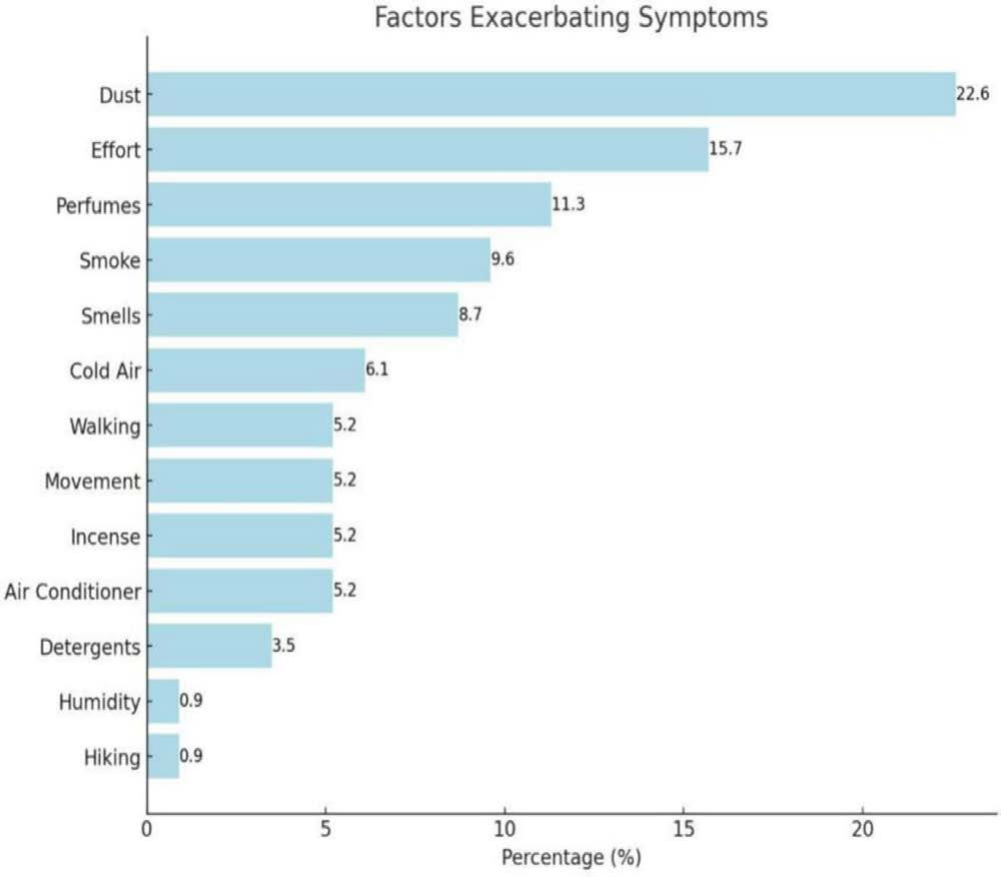
Environmental and physical factors reported to exacerbate nasal symptoms among pregnant participants.

In our study cohort, we found the prevalence of PR to be 29.8% (115 of 386 participants). PR most frequently occurred during the first trimester of pregnancy (47.8%), with 73% of the affected participants reporting moderate to severe symptoms, as assessed using the NOSE scoring system. Notably, 79.1% of the participants identified specific factors that exacerbated the rhinitis symptoms (Table 2). The most frequently reported triggers include dust, physical effort, olfactory triggers, such as perfumes and certain smells, and climatic changes (Fig. 1). In 80% of the cases, rhinitis symptoms persisted throughout pregnancy without resolution. Most participants did not seek medical consultations for PR. Instead, they opted for home-based alternative therapies to manage their conditions (Table 2).

**Table 2:** Prevalence and features of pregnancy rhinitis.

Parameter	Response	Frequency N (%)
Prevalence of PR among pregnant women	Yes	115/ 386 (29.79%)
	No	271/ 386 (70.21%)
PR experienced as per the trimester of pregnancy	First trimester	55/ 115 (47.83%)
	Second trimester	44/ 115 (38.26%)
	Third trimester	16/ 115 (13.91%)
Severity of PR as per the NOSE score	Mild	20/ 115 (17%)
	Moderate	52/ 115 (45.5%)
	Severe	33/ 115 (28.5%)
	Very severe	10/ 115 (9%)
Any exacerbating factors	No	24/ 115 (20.87%)
	Yes	91/ 115 (79.13%)
Occurrence of symptoms (as per trimesters)	First	20/ 115 (17.39%)
	Second	27/ 115 (23.48%)
	Third	38/ 115 (33.04%)
Resolution of symptoms (as per trimesters)	Didn’t disappear	92/ 115 (80.00%)
	First trimester	5/ 115 (4.35%)
	Second trimester	5/ 115 (4.35%)
	Third trimester	13/ 115 (11.30%)
Treatments chosen for rhinitis	Medical consultation	38/ 115 (33.04%)
	Alternative therapy	77/ 115 (66.96%)
Therapies used by participants	Nasal drops/spray	34/ 115 (29.79%)
	Home-based herbal remedies	81/ 115 (70.21%)

NOSE = Nasal Obstruction Symptom Evaluation.

Figure 1 illustrates the factors exacerbating symptoms associated with PR. Dust was the most common reported factor, followed by Efforts and perfumes.

**Figure 2.**
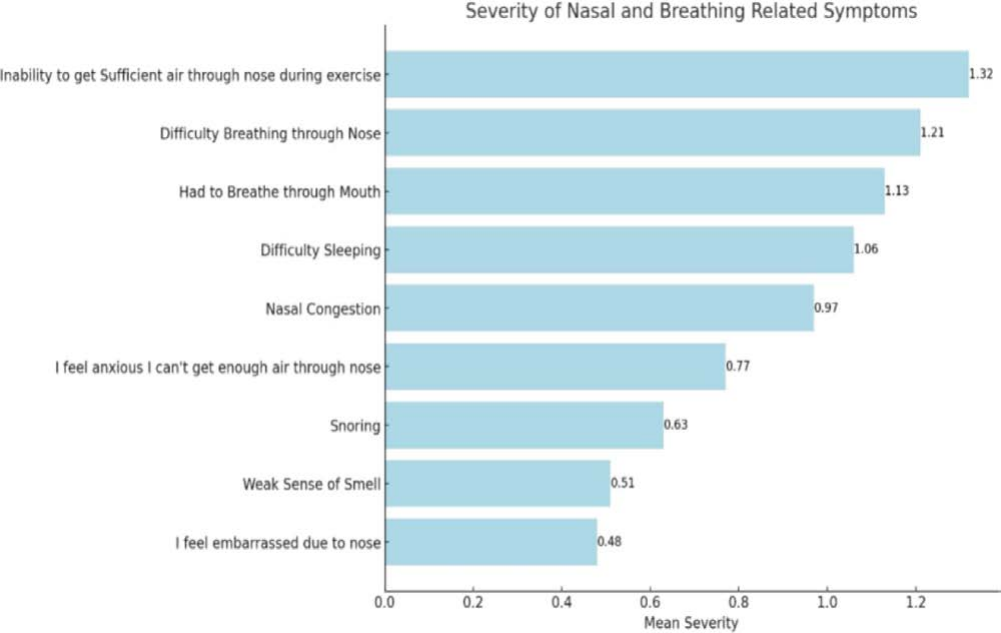
Mean severity scores of nasal and breathing-related symptoms reported during pregnancy.

We further analyzed the severity of different nasal symptoms associated with PR. Participants reported the following as the most severe symptoms: inability to inhale properly, compromised nasal breathing, increased oral breathing, insomnia due to a blocked nose, and nasal congestion (Fig. 2).

We further analyzed a subgroup of 115 participants with rhinitis to assess the correlation between rhinitis and various sociodemographic factors. We observed a comparable prevalence of rhinitis across different age groups. However, the prevalence declined significantly with increasing education level, from primary school through post-graduation (*P* < .001). Employed women had a significantly higher prevalence than their unemployed counterparts and students (*P* = .050). Chronic diseases significantly increased the prevalence of PR (*P* = .028); however, no single disorder had a significant individual impact. Additionally, none of the other demographic factors included in the study significantly affected the prevalence of PR in this cohort, as shown in Table 3.

**Table 3:** Association between PR and different sociodemographic and pregnancy-related features (n = 386).

Demographic factors	Categories	Frequency N (%) (pregnancy rhinitis absent)	Frequency N (%) (pregnancy rhinitis present)	*P* value
Age	18–25 yr	37 (80.4%)	9 (19.6%)	.4006
	26–30 yr	83 (67.5%)	41 (32.5%)	
	31–35 yr	78 (69.6%)	34 (30.4%)	
	>35 yr	73 (70.2%)	31 (29.8%)	
Education level	Uneducated	5 (100.0%)	0 (0.0%)	<.001
	Primary to middle	12 (34.3%)	23 (65.7%)	
	High school	109 (90.8%)	11 (9.2%)	
	Bachelor’s	131 (66.2%)	67 (33.8%)	
	Diploma	10 (41.7%)	14 (58.3%)	
	Postgraduate	4 (100.0%)	0 (0.0%)	
Career status	Unemployed	211 (71.5%)	84 (28.5%)	.050
	Employed	44 (60.3%)	29 (39.7%)	
	Student	14 (87.5%)	2 (12.5%)	
Monthly income	<5000 SAR	125 (69.8%)	54 (30.2%)	.6112
	5000–10,000 SAR	90 (73.2%)	33 (26.8%)	
	>10,000 SAR	56 (66.7%)	28 (33.3%)	
Smoker	No	267 (70.4%)	112 (29.6%)	.7295
	Yes	4 (57.1%)	3 (42.9%)	
BMI	Underweight	1 (33.3%)	2 (66.7%)	.6112
	Normal	64 (68.1%)	30 (31.9%)	
	Overweight	82 (73.2%)	30 (26.8%)	
	Obese Class 1	86 (70.5%)	37 (29.5%)	
	Obese Class 2	38 (70.4%)	16 (29.6%)	
Chronic disease	No	220 (73.1%)	81 (26.9%)	.02810
	Yes	51 (60.0%)	34 (40.0%)	
Different chronic diseases	Diabetes	22 (59.5%)	15 (40.5%)	
	Hypothyroidism	18 (69.2%)	8 (30.8%)	.632
	Anemia	4 (44.4%)	5 (55.6%)	
	Hypertension	4 (50.0%)	4 (50.0%)	
	Asthma	4 (50.0%)	4 (50.0%)	
No. of children	0	58 (75.3%)	19 (24.7%)	.0600
	1–3	146 (65.2%)	78 (34.8%)	
	4–7	64 (79.0%)	17 (21.0%)	
	>7	3 (60.0%)	2 (40.0%)	
Pregnancy type	Single	265 (70.3%)	112 (29.7%)	1.000
	Twin	6 (66.7%)	3 (33.3%)	
No. of miscarriage	0	177 (68.3%)	82 (31.7%)	.3439
	1–2	71 (77.2%)	21 (22.8%)	
	3–4	17 (63.0%)	10 (37.0%)	
	≥5	6 (75%)	2 (25%)	

PR = Pregnancy rhinitis, SAR = Saudi Riyals.

Table 4 presents the associations between various demographic and clinical factors and the presence of PR (*P* < .05). Notably, there were statistically significant correlations between work status (OR = 1.837, 95% CI: 0.0764–2.192), normal BMI (OR = 2.360, 95% CI: 1.024–5.438), diabetes (OR = 4.015, 95% CI: 0.915–17.615), and lack of chronic illness (OR = 0.362, 95% CI: 0.176–0.751). There was a minimal predictive value for PR in this cohort, as other characteristics, such as age, education, smoking status, income, and the number of children or miscarriages, did not show significant correlations (*P* > .05).

**Table 4:** Association between the individuals’ characteristics and PR.

Demographic factors	(pregnancy rhinitis present) OR (95% CI)	*P* value
Age (>35 yr)		
18–25 yr	0.400 (0.142–1.128)	.083
26–30 yr	0.799 (0.398–1.603)	.527
31–35 yr	0.832 (0.423–1.636)	.595
Education (uneducated)		
Educated	-	.999
Career status (unemployed)		
Employed	1.837 (0.0764–2.192)	.040
Student	0.407 (0.124–3.359)	.296
Monthly income (>10,000 SAR)		
<5000 SAR	1.327 (0.672–2.621)	.414
5000–10,000 SAR	0.982 (0.502–1.921)	.958
Smoker (yes)		
No	0.646 (0.124–3.359)	.603
BMI (obese Class 2)		
Underweight	2.329 (0.963–5.629)	.060
Normal	2.360 (1.024–5.438)	.044
Overweight	1.418 (0.617–3.259)	.411
Obese Class 1	3.800 (0.417–34.646)	.237
Chronic disease (yes)		
No	0.362 (0.176–0.751)	.006
Different chronic diseases (yes)		
Diabetes	4.015 (0.915–17.615)	.042
Hypothyroidism	1.545 (0.295–8.084)	.567
Anemia	0.789 (0.157–3.978)	.774
Hypertension	0.605 (0.063–5.785)	.662
Asthma	0.893 (0.158–5.047)	.898
No. of children (>7)		
0	0.640 (0.078–5.237)	.678
1–3	0.856 (0.166–6.485)	.880
4–7	0.395 (0.051–3.035)	.372
Pregnancy type (twin)		
Single	1.031 (0.228–4.669)	.968
No. of miscarriage (≥5)		
0	0.640 (0.078–5.237)	.678
1–2	0.856 (0.113–6.485)	.880
3–4	0.395 (0.051–3.035)	.372

### 4. Discussion

This study provides fresh evidence for the prevalence of PR among Saudi women. Compared with the only previous Saudi-based study by Albahkaly et al,^[10]^ which included 260 women from Riyadh, our study analyzed a larger sample of 386 women from Medina. The reported prevalence of PR in our cohort was 29.8%, which closely aligned with the 31.2% reported by Albahkaly et al, suggesting a consistent national estimate of 29% to 31%.

In a thorough review of the literature on rhinitis in pregnancy, Caparroz et al emphasized the diagnostic ambiguity between gestational rhinitis and rhinitis that develops during pregnancy for various reasons.^[6]^ Their results confirm our findings that symptoms begin in the first trimester and last throughout pregnancy by highlighting the importance of hormonal alterations, including those involving estrogen and progesterone, in nasal mucosal edema and congestion. Grajczyk et al confirmed the major symptom burden of pregnancy-related rhinitis, acknowledged dependence on non-pharmacological management, and identified no significant adverse neonatal outcomes.^[12]^ Further, their population-based study observed a modest increase in cesarean delivery rates among women with PR, but no significant correlation between PR and poor infant outcomes. The significant symptom load and dependence on non-pharmacological therapy may be indicative of underlying concerns regarding fetal safety and intervention risks, even though our study did not directly evaluate delivery outcomes. According to our data, PR symptoms were most frequently reported in the first trimester (47.8%), and in 80% of the cases, they persisted throughout pregnancy. The findings of Al-Ani et al, who noted that PR symptoms frequently start early in pregnancy and may last until birth, are consistent with this pattern.^[13]^ Our findings are supported by the association between mucosal edema and increased nasal congestion and early pregnancy hormonal surges in estrogen and progesterone. Interestingly, our study participants demonstrated low symptom resolution, with only 11.3% reporting alleviation in the last trimester, despite several studies suggesting symptom resolution in the third trimester due to hormonal stabilization, such as Kumar et al. Regional environmental characteristics such as high dust exposure and climatic variability, which our participants often mentioned as exacerbating triggers, may be the cause of this disparity.^[14]^

According to the NOSE score, the majority of study participants had moderate to severe symptoms. Dumitru et al discovered that PR considerably impacts the quality of life, with many patients experiencing impaired functioning during the day and sleep disruptions. This severity profile was supported by their research.^[15]^ Additionally, our study found that common exacerbating factors included dust, fragrances, physical activity, and climate change. These results aligned with those of Iordache et al, who highlighted how environmental irritants and inflammatory mechanisms can exacerbate pregnancy-related nasal symptoms.^[16]^ Our population’s high frequency of dust-related triggers in our study population may reflect regional exposure patterns, especially in arid settings.

Statistically significant correlations were observed between PR and the presence of chronic diseases (*P* = .028) and education level (*P* < .001). PR was more frequently reported by women with lower educational attainment, a finding that might be related to differences in access to preventive treatment and health literacy. This is in line with a Saudi cohort study by Alnemare et al, which found a correlation between a higher prevalence of untreated nasal problems during pregnancy and poorer educational attainment.^[17]^

Alternative therapies were selected by most of the participants (66.9%), with herbal cures being the most popular. Im et al discovered that pregnant women frequently choose herbal or alternative remedies over pharmaceutical treatments, primarily due to concerns regarding fetal safety.^[18]^ The significance of minimal intervention and evidence-based safety in therapy selection was also underlined by Caparroz et al, highlighting the necessity for more precise clinical recommendations and patient education.^[6]^

Although there are several limitations, this study provides insightful information about pregnant rhinitis (PR). First, participants may have misremembered or misclassified nasal symptoms because of their dependence on self-reported symptoms, which introduces recall bias. Furthermore, PR was not objectively identified owing to the lack of clinical confirmation, which increases the risk of misdiagnosing other types of rhinitis. Because the study included only 2 hospitals, its findings may not be representative of larger regional or national trends, further limiting their generalizability. Future studies should use clinical evaluations and objective diagnostic instruments to confirm PR and distinguish it from other nasal disorders, thereby overcoming these limitations. Including more healthcare facilities in various geographical areas would increase the representativeness of this study and provide a more thorough examination.

## 5. Conclusion

Our study provides invaluable insights into the prevalence of PR (PR) among Saudi women, highlighting a significant gap in existing regional data. These findings suggest that PR is more prevalent than previously understood and is often present in moderate-to-severe forms. Further research is essential to explore the observed correlations, such as the reduced frequency of PR among women with higher education levels, and the increased prevalence among employed women and those with chronic health conditions. To address the issue of underdiagnosis and underreporting, obstetrician-gynecologists (OB-GYNs) must collaborate with ENT specialists to assess nasal symptoms early in pregnancy and ensure that patients receive comprehensive education about PR. While this study contributes valuable knowledge on the clinical presentation and prevalence of PR, the results should be interpreted with caution owing to methodological limitations, including the potential influence of confounding factors. Future studies with more robust designs are necessary to confirm these associations and to enhance clinical practice.

## Author contributions

**Conceptualization:** Hanan I. Almuzaini, Rasil H. Almughamisi.

**Data curation:** Aseel A. Alamri, Thikra A. Alsenani, Maram A. Alhazmi.

**Formal analysis:** Rasil H. Almughamisi, Shahad A. Aljohani, Danah A. Alnajjar.

**Methodology:** Shahad A. Aljohani, Danah A. Alnajjar, Aseel A. Alamri.

**Supervision:** Hanan I. Almuzaini, Samah O. Alfahl.

**Writing – original draft:** Rasil H. Almughamisi, Shahad A. Aljohani, Danah A. Alnajjar, Aseel A. Alamri.

**Writing – review & editing:** Hanan I. Almuzaini, Rasil H. Almughamisi, Thikra A. Alsenani, Maram A. Alhazmi, Samah O. Alfahl.
